# Identification of quality and safety concerns in AI chatbot responses to procedural sedation: a comparative evaluation of ChatGPT, Gemini, and Copilot

**DOI:** 10.3389/frhs.2026.1830227

**Published:** 2026-05-08

**Authors:** Hüseyin Babun, Fatih Oluş

**Affiliations:** Department of Oral and Maxillofacial Surgery, Faculty of Dentistry, Akdeniz University, Antalya, Türkiye

**Keywords:** artificial intelligence, chatbots, confabulation, patient education, procedural sedation

## Abstract

**Introduction:**

Artificial intelligence–based chatbots are increasingly used by patients to obtain medical information before healthcare encounters. However, the reliability and safety of chatbot-generated responses remain uncertain, particularly for topics involving procedural sedation.

**Methods:**

This study evaluated the quality, safety, and confabulation risk of responses generated by ChatGPT, Gemini, and Copilot to common patient questions about procedural sedation. A set of standardized patient-oriented questions was submitted to each chatbot, and responses were independently evaluated by clinical experts using predefined criteria assessing informational quality, clinical safety, and the presence of confabulated or misleading content.

**Results:**

The results demonstrated variability in response quality across chatbots, with several answers containing incomplete information, safety omissions, or potentially misleading statements. Although many responses provided generally understandable explanations, important clinical details relevant to patient safety were inconsistently addressed.

**Discussion:**

These findings suggest that while AI chatbots may support patient education, their responses regarding procedural sedation may contain safety gaps and confabulated content that limit their reliability as standalone sources of medical information. Careful oversight and clinician-guided use of AI-generated health information may therefore be necessary to ensure safe and accurate patient communication.

## 1 Introduction

Sedation is widely used in dentistry and ambulatory medical procedures to reduce anxiety, facilitate patient cooperation, and improve procedural comfort. Unlike general anesthesia, procedural or conscious sedation allows patients to maintain spontaneous ventilation, preserve protective airway reflexes, and remain responsive to verbal or light tactile stimulation while experiencing reduced anxiety and discomfort. Because patients remain partially aware during sedation, effective preoperative counseling is essential to ensure understanding of the procedure, expected sensations, potential risks, intra-procedural monitoring, and postoperative recovery (1).

Preprocedural anxiety is a well-recognized challenge in dental and procedural sedation settings. Patients frequently report concerns related to pain, loss of control, complications, intraoperative awareness, and recovery. Dental fear and anxiety are known barriers to care, and inadequate preoperative information may further increase distress and reduce cooperation (2, 3). Consequently, structured and clear patient education is a fundamental component of safe and effective sedation practice.

In recent years, patients have increasingly turned to digital platforms to obtain medical information before undergoing procedures. Among these, artificial intelligence (AI)-based chatbots powered by large language models (LLMs) have emerged as widely accessible tools capable of generating conversational responses to health-related questions. These systems are able to encode substantial clinical knowledge and produce medically relevant responses, highlighting their potential role in healthcare information delivery (4). However, much of the existing literature has primarily focused on clinical accuracy and appropriateness, often based on physician evaluation, while comparatively fewer studies have examined how patients interpret, trust, and act upon chatbot-generated medical information. In addition, the rapid evolution of artificial intelligence systems means that existing evidence, although valuable, may not fully reflect the current capabilities and limitations of contemporary models, highlighting the need for ongoing and context-specific evaluation.

Recent patient-centered studies suggest that AI-generated responses are often perceived as understandable, accessible, and trustworthy, and may influence health-related decision-making. For example, simulated patient studies have demonstrated favorable ratings in communication quality and perceived empathy despite variability in clinical completeness (5). Similarly, readability-focused analyses have highlighted the importance of accessibility in shaping patient understanding (6). Systematic reviews further indicate that while LLMs can provide generally accurate information, concerns remain regarding incomplete responses and their potential impact on patient comprehension and safety (7). Domain-specific studies in areas such as emergency medicine and stroke care have also demonstrated variability in performance across different models (8).

Importantly, patient-centered evaluations and physician-based assessments represent distinct but complementary perspectives. While clinician evaluations provide insight into medical accuracy, patient-focused studies emphasize how information is perceived and used in decision-making. Despite this growing body of literature, the presence of safety-critical omissions—particularly in procedural contexts such as sedation—has not been systematically quantified. This represents an important gap, as incomplete information may be more clinically consequential than minor inaccuracies when patients rely on AI-generated content.

Procedural sedation is particularly sensitive to such limitations, as patient understanding directly influences preparation, cooperation, and safety. Essential counseling elements include fasting requirements, monitoring, airway considerations, postoperative restrictions, and recognition of warning symptoms. Omission or misrepresentation of such information may compromise patient safety and the overall effectiveness of preprocedural education.

Therefore, this study aimed to systematically evaluate not only the overall appropriateness of chatbot-generated responses but also the presence of safety-critical omissions and potentially misleading or unsupported content in the context of procedural sedation. By combining expert evaluation with a structured safety checklist and a dedicated reliability assessment framework, this study provides a clinically oriented and multidimensional analysis of chatbot performance, contributing to the growing literature on the reliability of AI-generated medical information.

## 2 Materials and methods

### 2.1 Study design

This cross-sectional comparative study evaluated the accuracy, clinical appropriateness, and safety-related content of responses generated by large language model (LLM)–based artificial intelligence chatbots—ChatGPT (OpenAI), Gemini (Google), and Copilot (Microsoft)—to commonly asked patient questions regarding procedural sedation. The study was conducted between January and February 2025 using a predefined protocol for question development, chatbot querying, expert evaluation, and safety–reliability assessment.

### 2.2 Question development

A total of 30 questions related to procedural sedation were developed by two anesthesiologists with clinical experience in dental and ambulatory sedation practices. The questions were designed to reflect common concerns expressed by patients during routine preoperative counseling. The selection of questions was informed by commonly reported concerns in the literature on dental anxiety, patient counseling, and sedation education (2, 9). To ensure comprehensive coverage of sedation-related information, the questions were categorized into four domains:

Definition and Procedure of Sedation (10 questions)
Safety, Monitoring, and Risks (10 questions)
Psychological Comfort and Control (4 questions)
Postoperative Recovery and Daily Activities (6 questions)

These domains were intended to represent the full spectrum of information typically addressed during preoperative sedation education. The final set of questions was reviewed and agreed upon by the two investigators to ensure clarity and relevance to typical patient inquiries. The complete list of questions used in this study is provided in Supplementary Table S1.

### 2.3 AI platforms and query procedure

Three publicly accessible AI chatbot platforms were included in the study:

ChatGPT (OpenAI)
Gemini (Google)
Copilot (Microsoft)

ChatGPT (OpenAI), Gemini (Google), and Copilot (Microsoft) were accessed using their publicly available free versions with default settings at the time of the study. No premium subscriptions, external plugins, or specialized prompt engineering techniques were used in order to simulate typical patient use conditions.

Each of the 30 questions was entered individually into the chatbot interfaces between January 15 and January 17, 2025. The exact wording of each question was used consistently across all platforms. No prompt optimization, follow-up prompts, or clarifying inputs were used in order to simulate typical real-world patient interactions. Responses were recorded immediately after generation and transferred into a structured dataset for analysis. Only the first complete response generated by each chatbot for each question was recorded and analyzed in order to reflect a typical single-query patient interaction. All chatbot queries were performed within the same time period to minimize potential variability related to time-dependent model updates.

To improve transparency regarding underlying model versions, additional investigation was performed using system-provided descriptions, direct queries to the chatbots, and publicly available documentation corresponding to the study period.

Based on these sources, Google Gemini indicated that it was operating on a Gemini 1.5 Flash–type model during the study period. ChatGPT was accessed via the publicly available free interface, which at that time was generally powered by a GPT-4–class model (e.g., GPT-4o or related variants), although the exact model version was not explicitly disclosed to users. Similarly, Microsoft Copilot did not provide explicit information regarding the underlying model version, and such details were not publicly available.

### 2.4 Expert evaluation of chatbot responses

A panel of ten anesthesiologists with at least five years of clinical experience in sedation practices independently evaluated all chatbot-generated responses. The use of a ten-member expert panel was intended to ensure diverse clinical perspectives while maintaining feasibility for detailed manual scoring, and is consistent with previous studies evaluating AI-generated medical responses (8, 10). To minimize bias, responses were anonymized and randomized prior to evaluation. Chatbot identifiers (ChatGPT, Gemini, or Copilot) were removed, and evaluators were blinded to the source of each response. This procedure was implemented to reduce potential evaluation bias and ensure objective assessment of response quality.

Each response was assessed using a five-point Likert scale based on its clinical appropriateness and accuracy:

1 = Very inappropriate
2 = Inappropriate
3 = Partially appropriate
4 = Appropriate
5 = Highly appropriate

For each response, the mean score across the ten evaluators was calculated and used as the primary measure of response quality. Internal consistency among the expert evaluators was assessed using Cronbach's alpha coefficient.

### 2.5 Safety-critical information assessment

In addition to expert scoring, chatbot responses were evaluated for the presence of essential safety-related information relevant to procedural sedation. A structured safety-critical checklist consisting of ten predefined domains was developed based on established clinical recommendations for sedation practice. The checklist items were defined based on commonly recommended elements of preoperative sedation counseling reported in the literature and clinical practice guidelines. The checklist included the following items:

Fasting instructions prior to sedation
Requirement for a responsible escort
Post-sedation driving restrictions
Potential respiratory depression risks
Monitoring of vital signs during sedation
Airway management or oxygen support considerations
Common adverse effects of sedation
Consideration of comorbidities or medication interactions
Warning signs requiring medical attention
Recommendation for clinician supervision or professional consultation

Each item was scored using a binary system:

  1 = information present

  0 = information absent

For each chatbot response, a safety omission rate was calculated based on the proportion of checklist items not addressed in the response.

### 2.6 Confabulation assessment

To evaluate the factual reliability of chatbot-generated responses, outputs were assessed for the presence of confabulations (i.e., fabricated or unsupported statements). Confabulations were defined as statements that were inaccurate, lacked clinical support, or had the potential to mislead patients.

Confabulation severity was categorized using a four-level scale:

0 = No confabulation
1 = Minor overgeneralization or incomplete statement
2 = Moderate unsupported claim or partially misleading information
3 = Major clinically misleading or incorrect information

Two independent reviewers evaluated confabulation severity for all responses using this predefined framework. Disagreements between reviewers were resolved through discussion and consensus.

### 2.7 Inter-rater reliability

Agreement between the two independent reviewers responsible for safety checklist scoring and confabulation assessment was evaluated using Cohen's kappa statistics. Kappa values were interpreted according to commonly accepted thresholds for inter-rater agreement.

### 2.8 Statistical analysis

Statistical analyses were performed using Python (version 3.11) with the NumPy, SciPy, and Pandas libraries. All analyses were conducted using pre-specified evaluation criteria defined before data analysis. For each chatbot, mean appropriateness scores were calculated for individual questions, overall performance, and each domain category.

Differences among chatbot platforms were analyzed using one-way analysis of variance (ANOVA). When statistically significant differences were identified, Tukey's honestly significant difference (HSD) *post-hoc* test was used for pairwise comparisons.

Safety omission rates and confabulation severity distributions were analyzed descriptively and compared across chatbot platforms when applicable. A *P*-value < 0.05 was considered statistically significant for all analyses. Although Likert scale data are ordinal in nature, parametric tests such as ANOVA were used based on the aggregation of scores across multiple independent raters, which allows approximation to interval-level data. This approach has been widely adopted in similar studies evaluating AI-generated medical responses. Nevertheless, the results were interpreted with consideration of this methodological limitation.

### 2.9 Ethical considerations

This study analyzed publicly accessible chatbot outputs and did not involve patients, patient data, or clinical interventions. The expert evaluators only assessed anonymized AI-generated responses. According to the research ethics policies of Akdeniz University, studies that do not involve human participants or identifiable personal data do not require Institutional Review Board (IRB) approval.

## 3 Results

### 3.1 Overall expert evaluation of chatbot responses

Each of the 30 sedation-related questions was answered by all three AI chatbots, resulting in a total of 90 responses. Each response was independently evaluated by 10 anesthesiologists using a 5-point Likert scale.

Gemini achieved the highest overall mean appropriateness score (4.73 ± 0.16, mean ± SD), followed by Copilot (4.01 ± 0.26, mean ± SD), whereas ChatGPT received the lowest mean score (2.89 ± 0.17, mean ± SD) (Table 1).

**Table 1:** Overall appropriateness scores of chatbot responses.

Platform	Mean score	SD	Min	Max	Ranking
ChatGPT	2.89	0.17	2.57	3.23	3rd
Gemini	4.73	0.16	4.40	4.97	1st
Copilot	4.01	0.26	3.50	4.47	2nd

**Figure 1:**
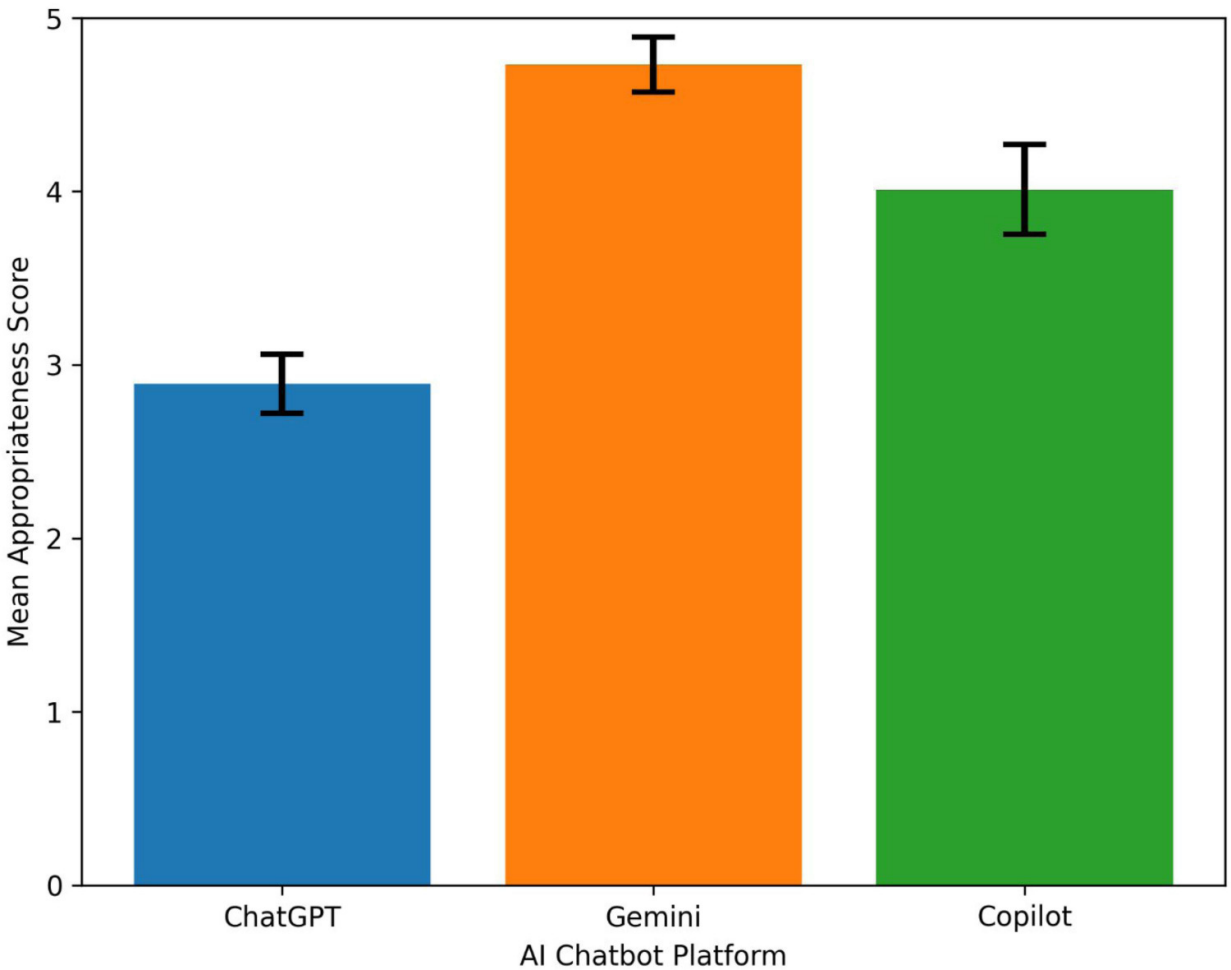
Overall appropriateness scores of AI chatbots. Bar graph illustrating the mean appropriateness scores of responses generated by ChatGPT, Gemini, and Copilot based on expert evaluation. Error bars represent standard deviation. Distinct colors are used to differentiate chatbot platforms and facilitate visual comparison.

The distribution of appropriateness scores across all questions is illustrated in Figure 1. The relatively lower performance of ChatGPT compared to the other platforms was notable. This difference may be related to variations in response style, including shorter or less detailed explanations, as well as differences in content filtering and response generation strategies across platforms.

### 3.2 Domain-Level performance

Mean scores for each chatbot across the four predefined question domains are summarized in Table 2.

**Table 2:** Mean appropriateness scores of chatbot responses across sedation question domains.

Domain	ChatGPT	Gemini	Copilot
Definition & Procedure	2.86	4.64	3.94
Safety & Monitoring	2.83	4.72	3.89
Psychological Comfort	3.03	4.80	4.28
Recovery & Daily Life	2.93	4.87	4.15

#### 3.2.1 Definition and procedure of sedation (questions 1–10)

Gemini provided the highest mean score in this domain (4.64), followed by Copilot (3.94) and ChatGPT (2.86).

#### 3.2.2 Safety, monitoring, and risks (questions 11–20)

Gemini again demonstrated the highest performance (4.72), whereas Copilot and ChatGPT achieved mean scores of 3.89 and 2.83, respectively.

#### 3.2.3 Psychological comfort and control (questions 21–24)

In questions addressing patient anxiety and intra-procedural awareness, Gemini obtained the highest mean score (4.80), followed by Copilot (4.28) and ChatGPT (3.03).

#### 3.2.4 Postoperative recovery and daily activities (questions 25–30)

Gemini achieved the highest mean score in this domain (4.87), followed by Copilot (4.15) and ChatGPT (2.93).

**Figure 2:**
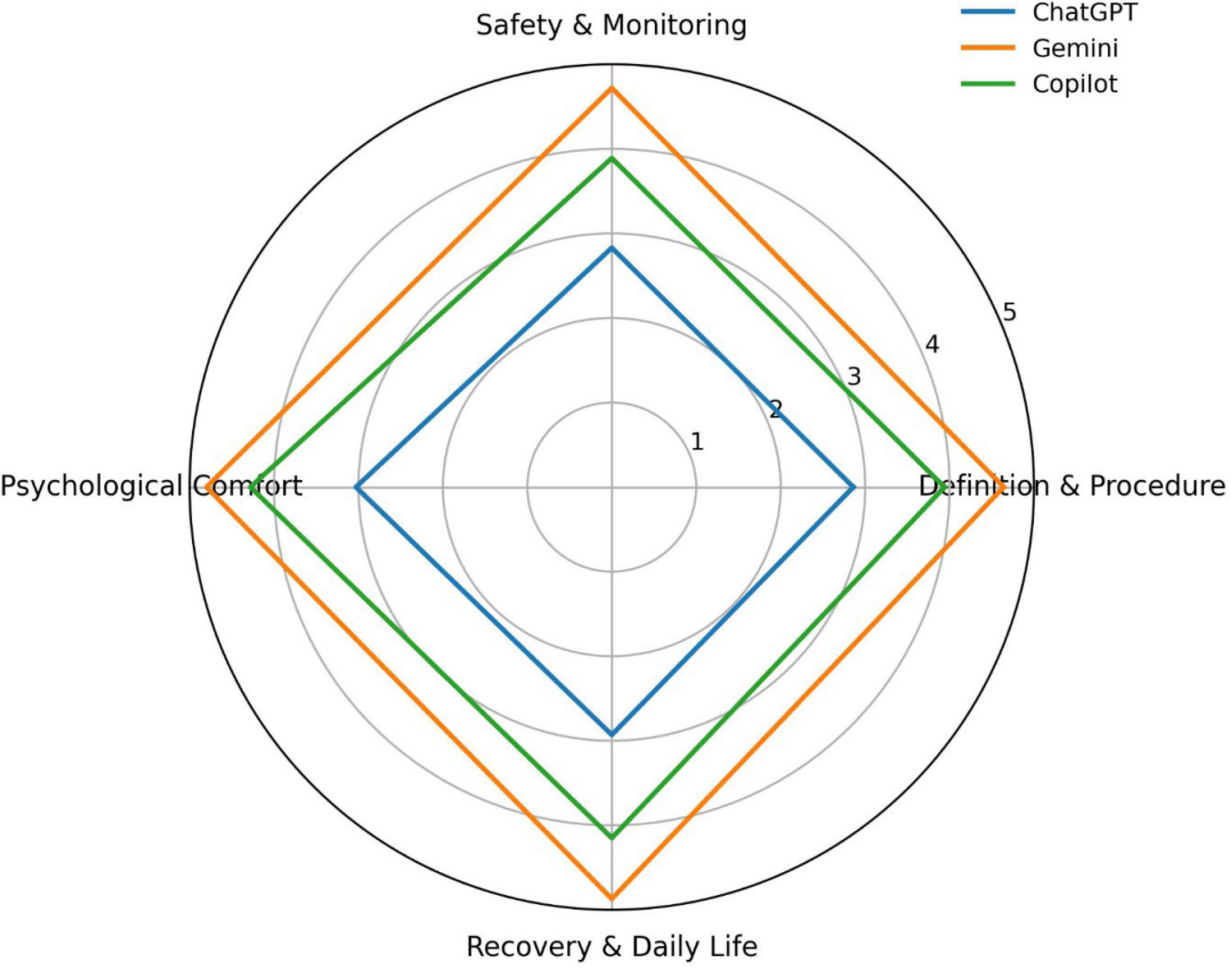
Radar plot comparison of domain-level performance of AI chatbots. Radar plot illustrating the comparative performance of ChatGPT, Gemini, and Copilot across four sedation-related domains: Definition and Procedure, Safety and Monitoring, Psychological Comfort, and Postoperative Recovery. This visualization highlights multidimensional differences in chatbot response quality.

A visual comparison of domain-specific performance is presented in Figure 2.

### 3.3 Comparison between chatbot platforms

A one-way analysis of variance revealed a statistically significant difference in mean appropriateness scores among the three chatbots [F (2, 87) = 629.80, *P* < 0.001].

Tukey's *post-hoc* analysis confirmed that all pairwise comparisons were statistically significant (*P* < 0.001 for ChatGPT vs. Gemini, ChatGPT vs. Copilot, and Gemini vs. Copilot), indicating meaningful differences in the clinical quality of responses generated by each platform. Detailed statistical comparisons are presented in Table 3.

**Table 3:** ANOVA and *post-hoc* comparison of chatbot appropriateness scores.

Comparison/Test	Result
One-way ANOVA	F(2,87) = 629.80, *P* < 0.001
Tukey *post-hoc* (ChatGPT vs. Gemini)	*P* < 0.001
Tukey *post-hoc* (ChatGPT vs. Copilot)	*P* < 0.001
Tukey *post-hoc* (Gemini vs. Copilot)	*P* < 0.001

### 3.4 Safety-critical information coverage

Evaluation of the safety-critical checklist revealed substantial variability in the inclusion of essential procedural sedation information across chatbot responses. Overall, Gemini demonstrated the highest safety completeness, whereas ChatGPT showed the highest omission rate of safety-related information.

**Figure 3:**
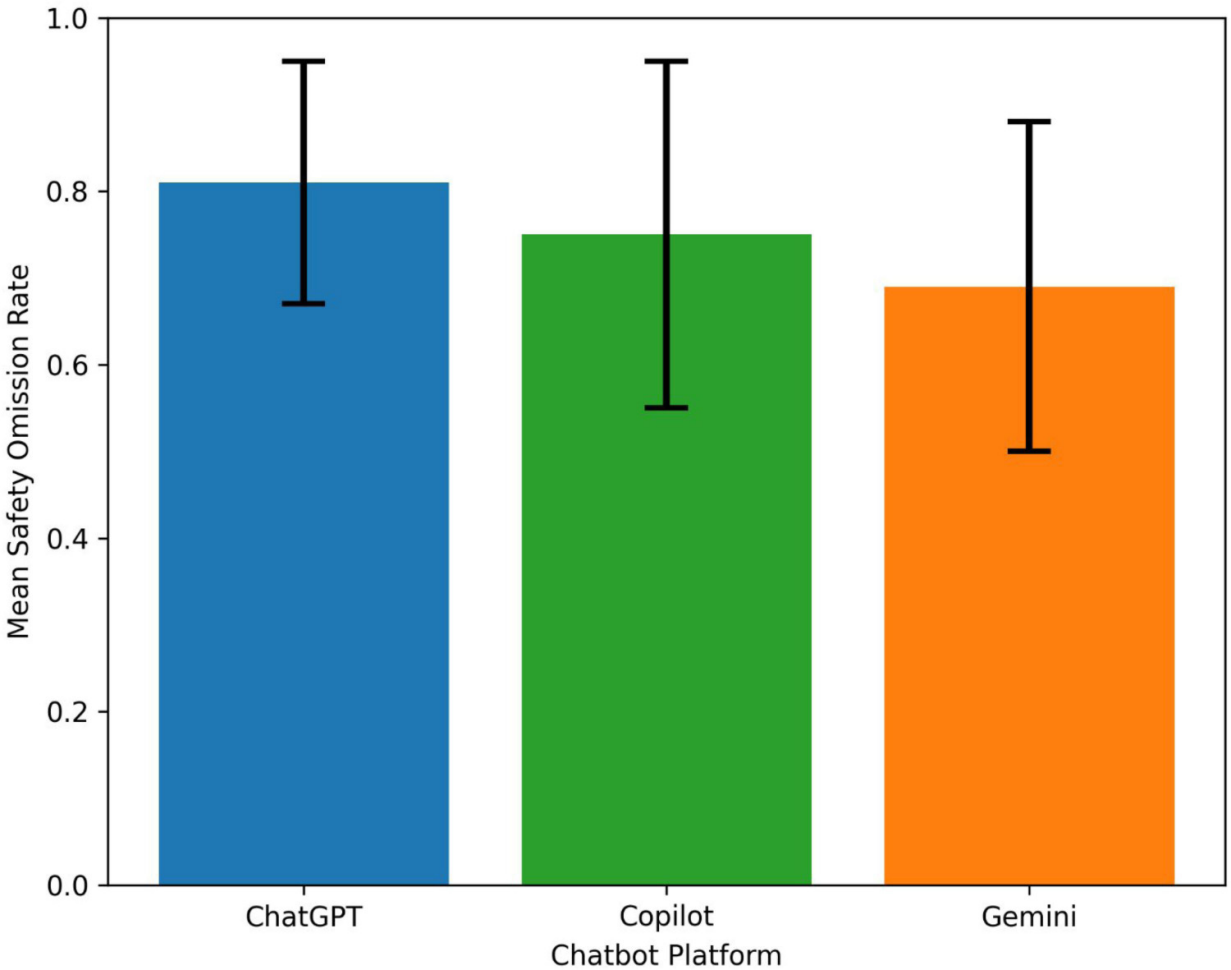
Safety omission rates across chatbot platforms. Bar graph showing the mean safety omission rates for responses generated by ChatGPT, Copilot, and Gemini. Error bars represent standard deviation. Distinct colors are used to differentiate chatbot platforms and enhance interpretability.

The mean safety omission rate across all responses was 0.75 ± 0.19, indicating that a large proportion of essential safety elements were not addressed in many chatbot-generated answers. Platform-specific omission rates are presented in Table 4, and a visual comparison of omission rates across chatbot platforms is shown in Figure 3.

**Table 4:** Safety omission rates across chatbot platforms.

Platform	Mean omission rate	SD
ChatGPT	0.81	0.14
Copilot	0.75	0.20
Gemini	0.69	0.19
Overall	0.75	0.19

Item-level analysis of the safety checklist revealed that several key elements of procedural sedation counseling were frequently omitted across chatbot responses. The most commonly missing information included fasting requirements prior to sedation, airway or oxygen support considerations, and warning signs requiring medical attention after sedation.

Other important safety elements—such as the need for a responsible escort, postoperative driving restrictions, and potential respiratory depression risks—were also frequently absent from chatbot responses. The high omission rate observed across chatbot responses may be partly influenced by the strict definition of safety-critical elements, which required explicit mention of each checklist item within a single response. As such, even partially informative responses were classified as containing omissions if specific elements were not directly addressed.

### 3.5 Confabulation assessment

Confabulation analysis revealed that the majority of chatbot responses contained minor factual inconsistencies rather than clinically misleading information. Overall, 4.4% of responses contained no confabulations, whereas 93.3% were classified as minor confabulations, typically involving overgeneralized or incomplete statements regarding sedation procedures. Moderate confabulations were identified in 2.2% of responses, while no major clinically misleading confabulations were detected.

The distribution of confabulation severity across all chatbot responses is summarized in Table 5.

**Table 5:** Confabulation distribution.

Confabulation Severity	Description	*n*	%
0	No confabulation	4	4.4
1	Minor overgeneralization	84	93.3
2	Moderate unsupported claim	2	2.2
3	Major clinically misleading	0	0

The high prevalence of minor confabulations should be interpreted in the context of the classification criteria used in this study. Minor confabulations included responses that were generally accurate but contained simplified, incomplete, or overgeneralized statements, which may have contributed to their high frequency.

### 3.6 Inter-rater reliability

Inter-rater reliability analysis demonstrated high agreement between the two independent reviewers for the safety-critical checklist. Cohen's kappa values for individual safety items ranged from 0.79 to 1.00, with perfect agreement observed for pre-procedural fasting instructions, requirement for a responsible escort, and post-sedation driving restrictions. When all safety checklist items were considered together, the pooled Cohen's kappa was 0.90, indicating excellent agreement.

For confabulation severity scoring, inter-rater agreement was lower but remained acceptable. The weighted Cohen's kappa for confabulation severity was 0.67, with an observed agreement of 96.7%. Detailed inter-rater reliability coefficients for individual safety-critical checklist items and confabulation severity scoring are presented in Table 6.

**Table 6:** Inter-rater reliability of safety checklist and confabulation severity ratings.

Safety checklist item	Cohen's *κ*
Pre-procedural fasting instructions	1.00
Requirement for a responsible escort	1.00
Post-sedation driving restrictions	1.00
Risk of respiratory depression	0.90
Monitoring of vital signs during sedation	0.80
Airway or oxygen support considerations	0.83
Common side effects of sedation	0.96
Comorbidity or drug interaction warnings	0.79
Warning signs requiring medical attention	0.84
Recommendation to consult a clinician for medical decisions	0.84
Pooled safety checklist reliability	**0** **.** **90**
Confabulation severity (weighted κ)	**0**.**67**

## 4 Discussion

The present study evaluated the quality, safety completeness, and confabulation characteristics of AI chatbot responses to common patient questions regarding procedural sedation. Using expert evaluation by anesthesiologists combined with a safety-critical checklist and confabulation assessment framework, the study demonstrated substantial variability among chatbot platforms. Although chatbot responses were generally able to explain basic concepts of procedural sedation, important safety-related counseling elements were frequently omitted. In particular, fasting instructions, airway considerations, and warning signs requiring medical attention were inconsistently included in chatbot-generated responses. These findings suggest that while AI chatbots can provide accessible explanations of sedation concepts, their ability to consistently deliver comprehensive procedural safety information appears limited. Our findings are consistent with previous studies evaluating the performance of large language models in medical information delivery. Prior investigations have demonstrated that AI chatbots are capable of generating generally accurate explanations of medical concepts, although the completeness and reliability of the information may vary across different clinical domains (11). Similarly, studies examining chatbot responses to patient health questions have reported that AI-generated responses may be perceived as informative and accessible, sometimes even outperforming physician responses in communication quality (9). Evaluations of chatbot responses to physician-generated clinical questions have also shown that AI systems can provide generally accurate medical information in many contexts (10). However, these findings should be interpreted with caution, as accuracy alone does not necessarily reflect the completeness or safety of the information provided. In the context of procedural sedation, incomplete responses may have greater clinical implications than minor inaccuracies, particularly when patients rely on such information for decision-making.

However, growing evidence suggests that AI-generated medical explanations may lack critical clinical detail despite appearing fluent and authoritative to users. Systematic reviews have highlighted that large language models frequently produce incomplete explanations and may omit essential safety information in healthcare-related responses (12). Similarly, domain-specific evaluations of AI-generated medical responses have demonstrated variability in accuracy and reliability depending on the medical topic being addressed (8, 13). These observations are consistent with the findings of the present study, in which chatbot responses generally conveyed the overall concept of procedural sedation but often failed to consistently include safety-critical instructions. While previous studies have primarily focused on the accuracy and readability of AI-generated responses, the present findings highlight the importance of evaluating safety completeness as a distinct and clinically relevant dimension. Even when responses are broadly accurate, omission of key safety information may limit their clinical reliability (7).

The observed differences between chatbot platforms may reflect variations in model architecture, training data sources, and safety alignment strategies implemented by different developers. Large language models are trained on diverse datasets and optimized using different reinforcement learning and safety-filtering techniques, which may influence how medical information is generated and structured. These factors may partly explain why some chatbots provided more comprehensive explanations of procedural sedation concepts, whereas others omitted important safety-related counseling elements. In addition, differences in response prioritization strategies may contribute to these findings. Some models may favor brevity and readability, potentially at the expense of completeness, whereas others may generate more comprehensive responses that include a wider range of clinical details. In safety-sensitive contexts such as procedural sedation, this trade-off may significantly influence the clinical usefulness of the information provided.

The omission of key safety elements may be particularly relevant in procedural sedation settings. Unlike general anesthesia, procedural sedation requires active patient cooperation and clear understanding of pre-procedural preparation and post-procedural precautions. Counseling elements such as fasting requirements, monitoring during sedation, post-sedation driving restrictions, and recognition of warning symptoms are essential components of safe sedation practice. Failure to communicate these instructions adequately may result in misunderstandings that could potentially compromise patient safety. As patients increasingly turn to online sources for medical information, the reliability of AI-generated educational content becomes an important consideration in clinical communication. These elements reflect standard components of pre-procedural counseling recommended in sedation practice guidelines and anesthesia safety recommendations. From a clinical perspective, omissions of safety-critical information may be more consequential than minor factual inaccuracies. In procedural sedation, patient understanding of essential elements such as fasting requirements, monitoring, and postoperative restrictions directly influences safety and procedural outcomes.

The findings of this study therefore highlight the importance of maintaining clinician oversight when AI chatbots are used as informational tools in healthcare. Although AI-generated explanations may help patients access preliminary information about procedural sedation, they cannot substitute for structured clinician-led counseling. Chatbot-generated information should therefore be considered a supplementary educational resource that may support patient understanding while reinforcing the need for direct clinician–patient communication.

### 4.1 Clinical implications

The findings of this study suggest that AI chatbots may function as accessible preliminary educational tools for patients seeking information about procedural sedation. However, the frequent omission of safety-critical elements indicates that chatbot-generated information should not be used as a substitute for structured pre-procedural counseling. Clinicians should remain the primary source of patient education regarding sedation safety, while AI tools may serve as supplementary resources that support patient understanding.

This study has several strengths. First, the evaluation framework combined expert scoring with a structured safety-critical checklist and confabulation severity assessment, allowing a multidimensional evaluation of chatbot-generated responses. Second, responses from three widely used AI platforms were compared, enabling cross-platform assessment of chatbot performance. Third, the evaluation was conducted by experienced anesthesiologists familiar with sedation practice, ensuring clinically relevant interpretation of the responses.

Nevertheless, several limitations should be acknowledged. The study evaluated chatbot responses to a predefined set of patient questions, which may not capture the full range of inquiries encountered in clinical practice. In addition, chatbot responses may vary depending on prompt wording, model updates, and system versions, meaning that the findings represent chatbot performance at a specific point in time. A further limitation is that the underlying model versions used by these platforms could not be precisely determined or controlled. Although efforts were made to identify model characteristics through system-provided descriptions, direct queries to the chatbots, and publicly available documentation, these platforms dynamically deploy models that are not fully transparent to end users. Therefore, precise version-level reproducibility could not be ensured. Furthermore, although predefined categories were used, the evaluation of confabulation severity involves a degree of subjective interpretation. Additionally, the high prevalence of minor confabulations observed in this study may be influenced by the broad definition used, which included responses that were generally accurate but simplified or incomplete. While such responses may improve readability, they may also contribute to partial understanding in complex clinical contexts. Another limitation relates to the rapidly evolving nature of large language models. AI chatbot responses may change over time as models are updated or retrained. Therefore, the findings of this study represent the performance of the evaluated platforms during the specific study period and may not fully reflect future chatbot behavior. Additionally, the relatively high omission rate observed in this study may be partly influenced by the strict criteria used to define safety-critical elements, which required explicit inclusion of each item within a single response. Finally, this study assessed the informational content of chatbot responses but did not evaluate patient comprehension or behavioral outcomes associated with AI-generated information.

From a future perspective, the development of large LLMs specifically trained or fine-tuned on validated patient education materials, such as clinical guidelines or standardized patient information leaflets, may represent a promising approach to improving the reliability and safety of AI-generated medical information. Such targeted training could help ensure that essential safety elements are consistently included while preserving the accessibility and conversational strengths of generative AI systems. Integrating domain-specific, curated knowledge sources into LLM frameworks may therefore enhance their potential as supportive tools for patient education, particularly in safety-sensitive contexts such as procedural sedation.

Future studies should investigate how patients interpret and comprehend chatbot-generated medical explanations and whether AI-assisted educational tools can be safely integrated into clinical communication strategies for procedural sedation in real-world clinical settings.

Taken together, these findings indicate that while AI chatbots can support patient education about procedural sedation, they should currently be considered supplementary information tools rather than reliable sources for comprehensive safety counseling.

In conclusion, AI chatbots were generally able to provide understandable explanations of procedural sedation concepts. However, important safety-related counseling elements were frequently omitted. While chatbot-generated responses may serve as supplementary educational resources, they should not replace clinician-led counseling when discussing procedural sedation and patient safety.

## Data Availability

The raw data supporting the conclusions of this article will be made available by the authors, without undue reservation.

## Supplementary material

Supplementary Table S1 List of questions used for chatbot evaluation.
