# Supplementary material for: Identification of quality and safety concerns in AI chatbot responses to procedural sedation: a comparative evaluation of ChatGPT, Gemini, and Copilot
Source: Front Health Serv. 2026 May 8;6:1830227. doi: 10.3389/frhs.2026.1830227 (PMC13194365; doi:10.3389/frhs.2026.1830227)
Supplement: Supplementary Table S1 — List of questions used for chatbot evaluation. [file Table1.docx]

**Supplementary Table 1 – List of questions used for chatbot evaluation**

**A. Definition and Procedure of Sedation**

1. What is conscious sedation, and how does it differ from general anesthesia?

2. How is sedation administered — intravenously or through a mask?

3. Will local anesthesia also be used along with sedation?

4. Will I be completely asleep during sedation?

5. If I remain conscious, will I be able to hear everything?

6. Will I be able to talk during sedation?

7. Will I feel any pain while under sedation?

8. How long does it take for the sedation to take effect?

9. Will I wake up immediately after the procedure is finished?

10. Will I remember anything about the dental procedure after sedation?

________________________________________

**B. Safety, Monitoring, and Risks**

11. Is conscious sedation safe for dental treatment?

12. Will I continue to breathe on my own during sedation?

13. Will my breathing and heart rate be monitored?

14. Will monitoring devices be attached during sedation?

15. If the dental procedure takes longer than expected, will the sedation remain effective?

16. For how long can sedation safely be maintained?

17. What happens if I become aware or move during the procedure — is it dangerous?

18. Can allergic reactions or drug interactions occur?

19. Will I feel nauseated or vomit after sedation?

20. Will I feel dizzy or lightheaded after sedation?

________________________________________

**C. Psychological Comfort and Control**

21. Will sedation completely relax me and reduce my anxiety?

22. Will I lose control of myself under sedation?

23. If I panic during the procedure, will you notice it?

24. Will I say or do anything unnecessary or embarrassing while sedated?

________________________________________

**D. Postoperative Recovery and Daily Life**

25. Can I go home after sedation?

26. Should someone accompany me, or is it safe to go alone?

27. Can I drive after sedation?

28. When can I return to my daily activities?

29. What should I do if I experience nausea, headache, or fatigue after sedation?

30. How long after the effects of sedation wear off can I make important decisions (such as signing documents, financial transactions, or notary procedures)?
